# Identifying Corporate Ownership in Assisted Living: Linking Communities to Parent Companies

**DOI:** 10.1093/geroni/igaf122.1405

**Published:** 2025-12-31

**Authors:** Lindsey Smith, John Bowblis, Momotazur Rahman, Kali Thomas, Gauri Gadkari, Cassandra Hua, Yashaswini Singh, Leya Kurzhiparambil

**Affiliations:** Oregon Health & Science University, Portland, Oregon, United States; Miami University, Oxford, Ohio, United States; Brown University, Providence, Rhode Island, United States; Johns Hopkins University, Baltimore, Maryland, United States; Brown University, Providence, Rhode Island, United States; University of Massachusetts Lowell, Lowell, Massachusetts, United States; Brown University School of Public Health, Providence, Rhode Island, United States; University of Massachusetts Lowell, Lowell, Massachusetts, United States

## Abstract

Corporate ownership in assisted living (AL), including private equity investment, has drawn increasing attention due to potential implications for care quality, financial practices, and resident outcomes. However, ownership data for AL communities is complex and fragmented, as different companies often own the property, manage the AL, and operate the AL. This makes it difficult to study how corporate structures shape operations. We developed and validated a method for systematically linking AL communities to corporate owners, operators, and managers–resulting in the first database linking AL communities to corporate ownership structures. We compiled a dataset of 14,866 AL communities (25+ units) from 2023 state licensing data and merged it with business records from Data Axle (n = 181,797), the National Investment Center for Senior Housing (n = 20,805), and corporate hierarchies from Duns & Bradstreet (n = 19,955 businesses linked to 307 parent companies). Using natural language processing (TF-IDF, Levenshtein distance, Jaccard index) and geospatial methods (geocoding, spatial joins) we matched 97% of AL communities to business characteristics and 86% to corporate ownership. A manual review of a random 10% sample confirmed 98% match accuracy. This approach provides a systematic way to study private equity and other ownership trends in AL, improving data reliability and reproducibility. Our findings highlight the need for transparency in ownership data and offer groundwork for evaluating how financial structures affect AL communities on a national scale. These approaches may be useful for studying ownership in other healthcare settings and for policymakers considering approaches to oversight and regulation.

